# Early sample tagging and pooling enables simultaneous SARS-CoV-2 detection and variant sequencing

**DOI:** 10.1126/scitranslmed.abj2266

**Published:** 2021-11-03

**Authors:** Alon Chappleboim, Daphna Joseph-Strauss, Ayelet Rahat, Israa Sharkia, Miriam Adam, Daniel Kitsberg, Gavriel Fialkoff, Matan Lotem, Omer Gershon, Anna-Kristina Schmidtner, Esther Oiknine-Djian, Agnes Klochendler, Ronen Sadeh, Yuval Dor, Dana Wolf, Naomi Habib, Nir Friedman

**Affiliations:** 1 Alexander Silberman Institute of Life Science, Hebrew University of Jerusalem, Jerusalem 9190401, Israel.; 2 Rachel and Selim Benin School of Computer Science, Hebrew University of Jerusalem, Jerusalem 9190401, Israel.; 3 Edmond and Lily Safra Center for Brain Sciences, Hebrew University of Jerusalem, Jerusalem 9190401, Israel.; 4 Hadassah Hebrew University Medical Center, Jerusalem 9112001, Israel.; 5 Lautenberg Centre for Immunology and Cancer Research, IMRIC, Faculty of Medicine, Hebrew University of Jerusalem, Jerusalem 9112001, Israel.; 6 Department of Developmental Biology and Cancer Research, IMRIC, Faculty of Medicine, Hebrew University of Jerusalem, Jerusalem 9112001, Israel.

## Abstract

Most severe acute respiratory syndrome coronavirus 2 (SARS-CoV-2) diagnostic tests have relied on RNA extraction followed by reverse transcription quantitative polymerase chain reaction (RT-qPCR) assays. Whereas automation improved logistics and different pooling strategies increased testing capacity, highly multiplexed next-generation sequencing (NGS) diagnostics remain a largely untapped resource. NGS tests have the potential to markedly increase throughput while providing crucial SARS-CoV-2 variant information. Current NGS-based detection and genotyping assays for SARS-CoV-2 are costly, mostly due to parallel sample processing through multiple steps. Here, we have established ApharSeq, in which samples are barcoded in the lysis buffer and pooled before reverse transcription. We validated this assay by applying ApharSeq to more than 500 clinical samples from the Clinical Virology Laboratory at Hadassah hospital in a robotic workflow. The assay was linear across five orders of magnitude, and the limit of detection was Ct 33 (~1000 copies/ml, 95% sensitivity) with >99.5% specificity. ApharSeq provided targeted high-confidence genotype information due to unique molecular identifiers incorporated into this method. Because of early pooling, we were able to estimate a 10- to 100-fold reduction in labor, automated liquid handling, and reagent requirements in high-throughput settings compared to current testing methods. The protocol can be tailored to assay other host or pathogen RNA targets simultaneously. These results suggest that ApharSeq can be a promising tool for current and future mass diagnostic challenges.

## Multiplexed SARS-CoV-2 sequencing

Existing SARS-CoV-2 diagnostic tests use RNA extraction followed by reverse transcription quantitative polymerase chain reaction (RT-qPCR), which limit their ability to quantify and sequence multiple variants in a single test. Chappleboim *et al*. describe a multiplexed next-generation sequencing (NGS)–based method called ApharSeq in which samples are pooled early via hybridization of barcoded primers. This allows for hundreds of pooled samples to undergo multiplexed reverse transcription, PCR, and sequencing to detect and classify variant sequences with high sensitivity and negligible contamination. The ApharSeq method was validated on clinical samples, demonstrating that dozens of samples can be pooled early to markedly reduce costs while still providing per-sample variant information.

## Teaser

A sequencing-based detection and genotyping assay for SARS-CoV-2 is based on early sample pooling using barcoded oligo hybridization.

## INTRODUCTION

Current methods for severe acute respiratory syndrome coronavirus 2 (SARS-CoV-2) testing include a panel of reverse transcription quantitative polymerase chain reaction (RT-qPCR) tests, which are typically applied to nasopharyngeal swab samples (*1*). The swabs are mixed in a lysis buffer or heat-inactivated in a transport buffer, followed by RNA extraction and RT-qPCR. Samples with cycle threshold (Ct) lower than 35 are typically considered positive in these tests (*2*, *3*). Although these tests are sensitive and specific, access to qualified labor and specialized equipment and reagents have limited testing capacity at different stages of the coronavirus disease 2019 (COVID-19) pandemic (*4*, *5*). Testing capacity is also limited by tests that require samples to be treated as separate qPCRs with fixed reaction times. The most common strategy used to overcome this limitation has been sample pooling to achieve varying levels of “test compression,” but pooling strategies reduce the test sensitivity by diluting individual samples, and they rely on relatively low viral prevalence rates to be effective (*6*, *7*).

In the past decade, next-generation sequencing (NGS) has replaced RT-qPCR and microarrays as the assay of choice for quantifying RNA molecules in research. During the COVID-19 pandemic, different NGS-based assays have been proposed to measure the presence and abundance of the viral genome in samples (*8*–*16*). In addition to detection and quantification, these assays can provide near real-time sequence information and provide epidemiologists with data on the emergence of new variants (*17*, *18*). Similarly, by assaying the RNA from host cells, aspects of the immune response in infected individuals can be characterized (*19*) and can provide potential insights into the development of new treatments or vaccines.

Here, we propose an improved RNA sequencing (RNA-seq) protocol that allows for pooling of barcoded samples before RT, which we called amplicon pooling by hybridization and RNA-seq (ApharSeq). This workflow is relevant to large-scale testing by reducing labor, reagents, and overall costs by orders of magnitude in these settings.

We show that we can introduce barcoded and target-specific RT primers to the samples, allowing them to hybridize to target RNA molecules already in the lysis buffer or after an RNA cleanup step on polyT magnetic beads. Sample RNA is captured on beads after hybridization with the barcoded primers, and the primer-RNA hybrids are preserved during a subsequent wash step. The bead-bound RNA is isolated, and hundreds of samples are pooled to undergo RT from the primers that remain hybridized to their original targets. The pooled samples undergo library PCR and sequencing, and viral RNA genome counts per sample are determined by the sample-specific barcodes and unique molecular identifiers (UMIs) introduced at the beginning of the protocol. The observed molecules are also examined for known and unknown mutations. We validated our test on blinded synthetic samples and on a collection of ~550 clinical samples. We demonstrate that cross-sample contamination in this workflow is negligible, and we determined sensitivity to be ~800 to 1600 copies/ml, comparable to existing U.S. Food and Drug Administration (FDA)– and European Union (EU)–approved tests (*20*).

## RESULTS

### A simple and quick RNA capture step

SARS-CoV-2 nasopharyngeal swab samples typically arrive in lysis buffers that contain protein denaturation and degradation reagents. RNA extraction from lysis buffer is needed to allow for subsequent enzymatic reactions, including RT. The SARS-CoV-2 genome is a polyadenylated 30-kb RNA molecule. Thus, we tested, Solid Phase Reversible Immobilization (SPRI)- and polyT bead–based RNA capture techniques (*21*). These bead-based methods are inexpensive, rapid, and compatible with automation. In terms of RNA yield, the performance of both bead types was within a ±50% range of a widely used commercial kit (see supplementary note on RNA capture; Fig. 2A and fig. S1). Preliminary tests showed that both bead-based capture methods could be used with ApharSeq (fig. S1), highlighting the independence of the tagging step from the RNA capture technique. We focused on the polyT bead–based method, which can proceed without elution of RNA from the polyT beads, as the next steps of the protocol can be applied directly on bead-bound RNA.

### Barcoded primers added to lysed samples prime RT reactions

**Fig. 1.**
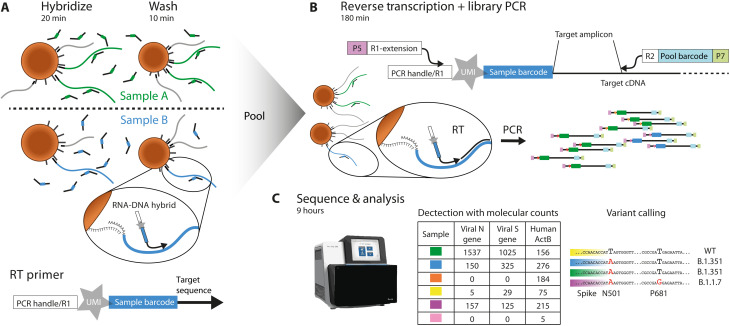
ApharSeq overview. (**A**) Barcoded and uniquely identifiable RT primers are hybridized to samples in transport/lysis buffer, and homemade paramagnetic polyT beads are used for a quick wash and buffer replacement step. (**B**) Beads are pooled and RNA undergoes an RT/PCR with prehybridized target-specific barcoded primers to generate a sequencing library. (**C**) Libraries are sequenced and analyzed, and PCR duplicates are collapsed to molecular counts for detection and further analysis (e.g., major variant calling by sequence analysis and contact tracing by minor variations).

We designed barcoded RT primers for the viral E gene (reverse), as it appears in the World Health Organization panel (*22*). The primer includes an interleaved 10–base pair (bp) barcode and a 10-bp UMI to allow for single-molecule counting (Fig. 1A, Materials and Methods, and supplementary note on primer design) (*23*). Each sample is hybridized to primers with a different barcode (Fig. 1A), effectively identifying the source of RNA molecules for the remainder of the process. The bead-bound RNA is washed, pooled with other samples, and reverse-transcribed to generate barcode-labeled complementary DNA (cDNA) copies.

To evaluate the efficacy of primer-RNA hybrid formation and stability through the cleanup stage, we designed a qPCR targeting the generic PCR handle on the RT primer and the amplicon target sequence. This assay allowed us to quantify the hybrids that survived wash steps and generated cDNA molecules. Using the qPCR assay, we established that RT primers remain hybridized during RNA capture and initiate RT reactions (fig. S2). We used this assay to run several optimizations for the first steps of the protocol and markedly improved the cDNA yield (fig. S2).

### Sequencing library preparation

**Fig. 2.**
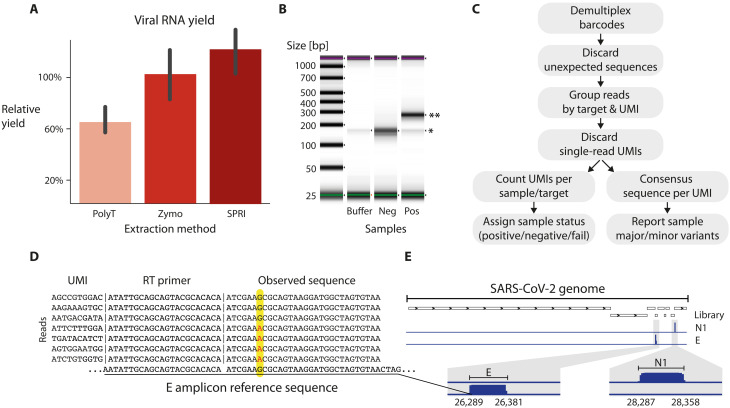
The ApharSeq pipeline generates specific sequencing libraries. (**A**) RNA capture yields by homemade polyT and SPRI paramagnetic beads is efficient (~60–120% compared to Zymo kit) and quick (<30 min for a 96 plate on a robotic system). (**B**) Sequencing libraries of the viral envelope amplicon (E) are specific to positive samples (*primer dimer, **expected amplicon: 269 bp). (**C**) Analysis outline: sequencing reads are demultiplexed by barcodes, compared to the expected amplicon sequence, grouped by UMI, and counted to estimate viral load. Observed sequences are used to call viral variants. (**D**) Observed sequences conform to the reference genome in >99.9% of reads. However, in at least one sample, we observe a sequence variation in reads from the E amplicon at 26,353 (G to A). Observed single-nucleotide polymorphism (SNP) is in more than 90% of reads of each UMI shown (~200 reads each), excluding the possibility of sequencing errors. (**E**) Genome browser view of N1 and E amplicon libraries highlights target specificity.

The next step in the ApharSeq protocol is to generate sequencing libraries. This is achieved in a single PCR step, amplifying the target amplicons using a combination of a generic primer targeting the tail of the RT primer and amplicon-specific primers (Fig. 1B). These primers introduce Illumina-compatible sequences flanking the target amplicons (Fig. 1B). We applied this PCR to positive and negative samples and consistently obtained amplicon-specific libraries only in SARS-CoV-2–positive samples (Fig. 2B). These libraries yield highly specific results, with >95% of reads aligning to expected viral target sequences in positive samples (Fig. 2C) upon sequencing on NGS platforms, and the remaining <5% of reads were mostly primer dimers.

### Cross-sample contamination is minimal

**Fig. 3.**
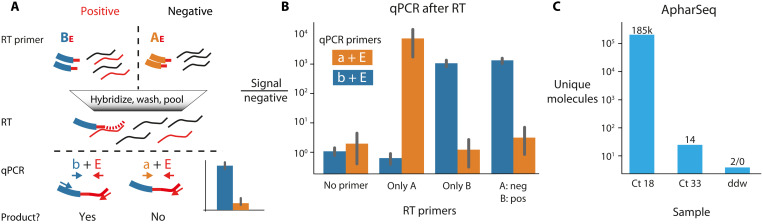
Minimal cross-contamination in ApharSeq. (**A**) qPCR cross-contamination assay: Different RT primers (uppercase AE/BE) targeting the viral E amplicon are hybridized to a positive/negative sample, then samples are pooled and reverse-transcribed, and RT primer–specific qPCR primers (lowercase a/b) are used in qPCR to detect successful RT reactions on the viral target. (**B**) qPCR results in fold change relative to no template control. (**C**) Minimal cross-contamination in ApharSeq sequencing libraries as quantified by unique molecules detected in a pool of four samples with Ct 18, Ct 33, and two negative controls (ddw). Numbers above the bars are the counts of unique molecules in each sample.

A critical concern related to pooling samples early in the protocol is that RNA molecules may be erroneously tagged due to residual free primers or other artifacts during RT, PCR, or sequencing (*24*). To test potential cross-contamination levels at the RT stage, we hybridized positive (Ct 26) and negative samples with two differently barcoded primers, pooled them, performed RT, and tested the amount of cross-contamination by barcode-specific qPCR (Fig. 3, A and B). We find that the pooled negative sample is indistinguishable from the unpooled negative sample, suggesting that cross-contamination is negligible.

To examine potential cross-contamination during PCR or sequencing (*25*), we subjected two samples with vastly different viral loads [high (Ct 18) or low (Ct 33)] and two negative controls (ddw) to ApharSeq. We hybridized the barcoded primers and pooled the samples before RT and PCR (Fig. 3C). Using the UMI sequence in each read, we were able to collapse PCR duplicates and provide an accurate and robust count of molecules captured in the assay. We found that barcodes that were hybridized to negative samples had at least 90,000-fold less observed molecules than those that were hybridized to the high Ct positive sample. These results are not unique to the polyT-based capture and were qualitatively replicated using the alternative SPRI-based RNA capture (fig. S1D). We conclude that cross-sample contamination is a minor issue in ApharSeq.

### ApharSeq is quantitative and sensitive

**Fig. 4.**
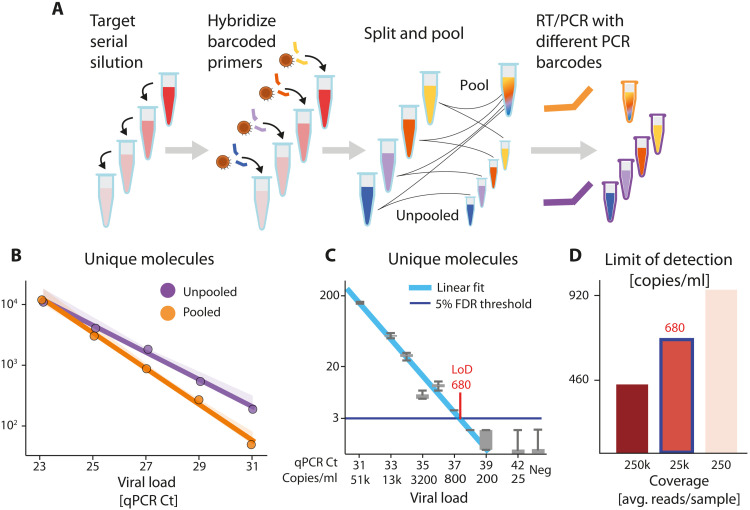
ApharSeq sensitivity estimation on added samples. (**A**) Target titration experimental scheme. SARS-CoV-2–positive sample is diluted in lysis buffer. Diluted samples are hybridized to barcoded ApharSeq primers (interior tube color) and split, so they can be assayed separately or pooled. Samples are subjected to PCR with different barcodes (purple/orange) to distinguish their treatments. (**B**) Assay is linear in pooled and unpooled ApharSeq assays (*P* < 0.001). The linearly extrapolated LoD for pooled samples is ~Ct 35.7 and is four times lower than the LoD for the single samples. (**C**) Low target titration experiment that includes addition of a viral target control for quantification. Gray boxes are molecular counts over read down-sampling replicates that show that the LoD is ~680 molecules/ml (Ct ~37.4). (**D**) LoD as a function of sequencing depth as derived by down-sampling the sequencing reads. Blue outlined bar (~25,000 reads per sample) corresponds to data in (C).

To evaluate the dynamic range of ApharSeq, we titrated a positive sample into lysis buffer and generated samples that spanned a 64-fold range (Ct 23 to 31). We applied ApharSeq to these samples in a pool and as individual samples (Fig. 4A). We found that the number of sequenced unique molecules scales linearly with the input (*P* < 0.001; Fig. 4B). By accounting for observed background in negative samples, we predict the limit of detection (LoD) to be ~Ct 35.3. We also compared the titration curve of the pooled and unpooled samples, which revealed minimal contamination between pooled samples (Fig. 4B).

We tested the LoD directly by performing another pooled titration experiment with highly diluted samples with a Ct range of 30 to 42 (Fig. 4C) and added samples with prequantified viral RNA (see Materials and Methods). This allowed us to estimate the end-to-end capture rate of ApharSeq at ~1.5% as we observed 33 and 14 molecules out of an input of ~2000 and ~1000 molecules, respectively. Similarly, we could calibrate the titration curve from Ct units to molecular counts and found the LoD to be 450 to 900 molecules/ml, depending on sequencing depth (Fig. 4D), threshold selection, and input volume used (see Materials and Methods).

### A multiple target assay

**Fig. 5.**
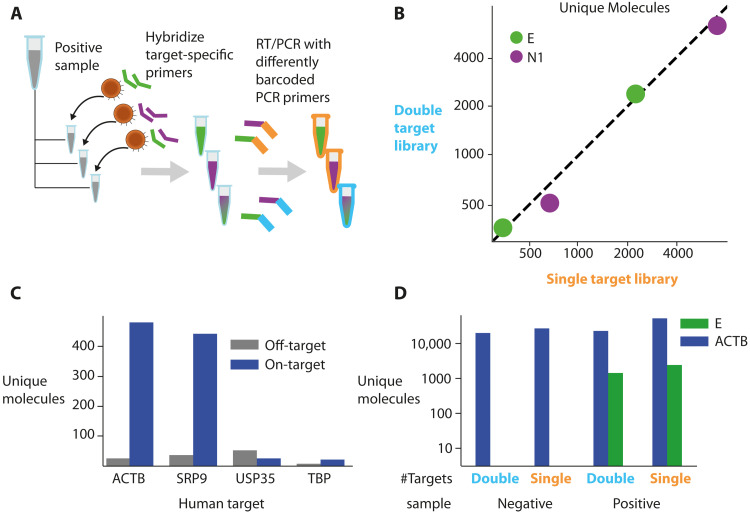
Multi-target ApharSeq libraries. (**A**) Multiple target assay scheme. A positive sample was split and hybridized with E, N1, or E and N1 RT primers. These samples underwent ApharSeq with differently barcoded PCR primers to delineate reads from the double/single hybridization conditions. (**B**) ApharSeq libraries for high/medium viral load samples for the N1 and E amplicon separately (*x* axis) yield similar counts to a single ApharSeq library for both targets (*y* axis). Units are unique molecular counts. (**C**) Human target tests for multiple genes on a pool of negative samples highlights potential specific targets at different expression levels (see all targets tested in fig. S3). (**D**) Human and viral targets multiplexed in the same ApharSeq library (“double”) allow for internal control in negative/positive samples. Units are unique molecular counts.

A major advantage of sequencing-based assays is their capacity to capture and read large numbers of targets from the same sample (*26*). We next examined the potential for a multi-target assay by multiplexing two targets. We designed RT and PCR primers for the viral N1 amplicon, as described in a CDC (Centers for Disease Control and Prevention) panel (*27*), and used these primers in conjunction with the E amplicon RT and PCR primers. We applied ApharSeq to a positive sample with each primer separately or with both primers together (Fig. 5A). The results of individual and multiplexed amplicons were almost identical (Fig. 5B), suggesting that the viral target sequences are amplified with minimal interactions and can be probed simultaneously to expand sequence information and improve confidence and sensitivity. The N1 amplicon yielded roughly two- to threefold more molecules than the E amplicon, consistent with previous reports (*28*, *29*).

As an internal control, we designed primers for several human transcripts with varying expression levels (*30*). After a preliminary test (Fig. 5C and fig. S3), we decided to continue with the *ACTB* amplicon as it is also used in an approved detection kit (*31*). We subjected positive and negative samples to the ApharSeq pipeline with primers targeting viral E and human *ACTB* amplicons to produce sequencing libraries, albeit with slightly reduced yields (Fig. 5D). qPCR tests on mixed libraries showed that decreasing the proportion of the human-specific primer in the PCR reduced the human/viral amplicon ratio accordingly, allowing for calibration of the number of reads allocated to each target in a multi-target library (fig. S3).

### Evaluation of clinical samples

Last, we validated that ApharSeq can be used at scale by evaluating hundreds of samples. We developed and tested a robotic protocol on a Tecan liquid handling station. With our current unoptimized protocol, a single 96-sample plate is processed for 40 min and can be pooled into a single tube for RT-PCR. We used 96 barcoded RT primer plates for the N1/E/ActB amplicons, with barcodes from a standard Illumina barcode collection.

**Fig. 6.**
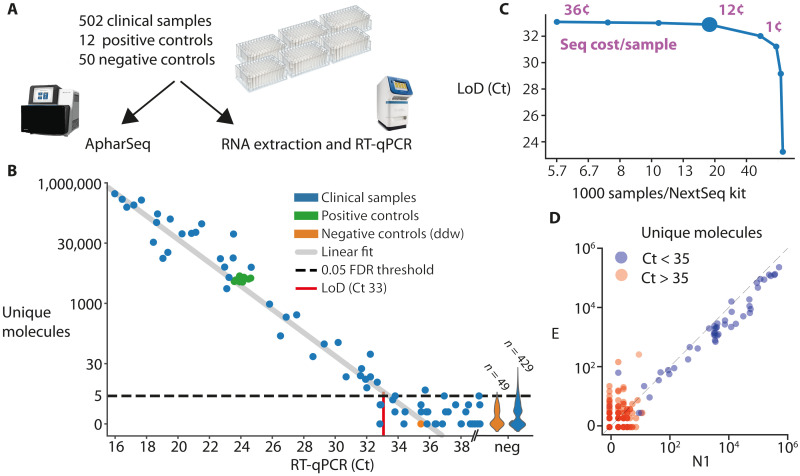
Robotic test of hundreds of clinical samples. (**A**) Experimental design for clinical samples. Samples were randomly assigned to 96-sample plates with ddw-negative controls in each plate and two standard positive controls derived from a clinical sample that was diluted in negative samples as to suffice for multiple tests. The plates were then split and subjected in parallel to ApharSeq or to the standard clinical pipeline at Hadassah hospital (Zymo RNA extraction and BGI RT-qPCR). (**B**) The number of unique N1 molecules observed in each sample (*y* axis) is plotted against the measured Ct for the same sample. LoD was determined by controlling the false-positive rate to be 5% and assuming Poisson noise. Violin plot on the right of the *x* axis depicts the distribution of unique molecules observed for the samples that were not detected in the RT-qPCR. Positive controls (green) highlight reproducibility between clinically distinct and varied pools. (**C**) Subsampling analysis of the sequencing data demonstrates that samples are sufficiently sequenced and that there is a minimal decrease in sensitivity (*y* axis) when sequencing depth decreases down to ~25,000 reads per sample, which is equivalent to ~16,000 samples in a single NextSeq run (larger blue marker). Purple numbers indicate the sequencing cost per sample in selected sequencing depths. (**D**) Viral amplicon correlation within samples. Unique molecules of the E (*y* axis) and N1 (*x* axis) amplicons are plotted per sample, demonstrating reproducibility. Color correlates to the Ct threshold currently used in the clinically approved protocol (pos Ct < 35).

We obtained positive (*n* = 37) and negative (*n* = 465) clinical nasopharyngeal swab samples. We randomly assigned samples to six 96-sample plates such that each plate had ~7.5% positivity rate (on average), and added positive and negative standards to each plate (Fig. 6A). The samples in each of these plates were divided in half into two identical plates; one plate underwent standard RNA extraction and quantification by RT-qPCR, and the other plate underwent the ApharSeq protocol (Fig. 6A).

The positive controls exhibited high reproducibility between plates (Fig. 6A), and we observed quantitative agreement between the human internal control in the RT-qPCR assay and our *ACTB* amplicon reads, with a comparable number of missing values in both assays (i.e., samples that will require retesting; fig. S7). We compared the Ct value of each sample to the amount of unique molecules and observed a strong linear agreement with the N1 amplicon (*R*^2^ = 0.95, *P* < 10^−38^), which further established the quantitative nature of the assay (Fig. 6B), and we also observed a strong correlation between the N1 and E amplicons. In concordance with other reports (*29*), we observed more unique molecules of the N1 amplicon in virtually all the samples (Fig. 6D). To determine the LoD of the assay, we first fit the negative samples with zero-inflated Poisson to find a threshold for the number of reads at which the false-positive rate is below 0.05%. We then fit a linear model to the positive samples and find the maximal Ct where 95% of positive samples would be above the threshold. This procedure estimated a LoD of Ct 33, which is equivalent to ~1000 copies/ml (Fig. 6B and Materials and Methods).

A subsampling analysis of the data showed that sensitivity is maintained down to a sequencing depth of ~25,000 reads per sample (Fig. 6C). Overall, we conclude that the robotic ApharSeq protocol works efficiently, with minimal cross-sample contamination, and is highly quantitative.

### Identifying viral variants in clinical samples with ApharSeq

**Fig. 7.**
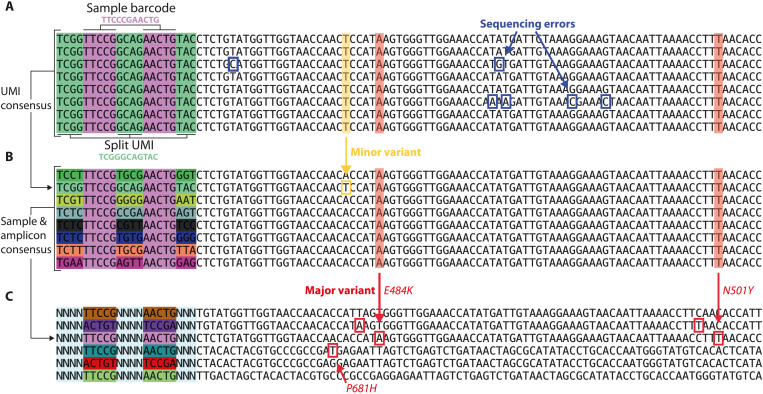
Using ApharSeq for common strain classification. (**A**) Raw reads with the same UMI. Sequencing errors are detected (blue boxed letters) when collecting all same UMI reads emanating from the same RNA molecule. (**B**) Variant calling using the UMIs from the same sample (sample barcode in light purple); different molecules can be distinguished, properly counted, and used for major (red highlight/box) and minor (yellow highlight/box) variant calling per sample [UMI displayed in (A) is an outlier with an A→T variation indicated in green]. (**C**) Major variants per sample are called with dozens to thousands of unique molecules collapsed to a single observed consensus sequence at each assayed amplicon. Red boxes indicate major variant discrepancies from the reference sequence. Different offset primers are used to increase signal complexity during sequencing. We observe known spike protein mutations (N501Y, P681H, and E484K) in some of the shown samples (*48*, *49*).

ApharSeq uses sequencing for the detection of viral molecules, and hence, it is well suited to variant calling. A common hurdle in amplicon-based genotyping assays, especially in the case of viruses that might manifest multiple minor variants in the same host, is the inability to distinguish between PCR and sequencing errors from underlying genotype variants (*32*, *33*). Unlike other common viral genome sequencing protocols (*34*, *35*), ApharSeq incorporates a UMI at the RT step. Because PCR duplicates are produced with high fidelity, it is unlikely that sequencing or PCR errors will introduce the same variation in multiple copies of the same original molecule. ApharSeq can detect multiple reads with the same UMI and filter technical errors according to their consensus (Fig. 7A). Thus, UMIs confer increased confidence in the observed sequence and enable identification of minor genetic variants in the sample (Fig. 7B).

As a proof of concept, we designed additional primers to target the area around three mutations (N501Y, E484K, and P681H) in the spike gene of the UK (B.1.1.7/alpha) and South-African (B.1.351/beta) variants (*36*, *37*). ApharSeq was able to detect these mutations when applied to positive samples suspected of being infected with these variants (Fig. 7C). Extrapolating from the number of observed molecules in these samples, we estimate that this amplicon around the P681 position in the spike gene will allow detection and variant calling up to Ct ~31.

## DISCUSSION

RT-qPCR assays constitute the testing backbone in the COVID-19 pandemic and remain a critical tool for this constantly changing pandemic. Some sample pooling strategies have proven to be useful (*6*, *7*), especially in light of shortages in reagents and testing equipment, but they remain limited when viral prevalence is high. RT-qPCR tests are not suited for genomic monitoring, and low- to mid-throughput NGS assays are currently used for the detection of existing and emerging SARS-CoV-2 variants of concern.

Improvements in NGS methods in the last decade have revolutionized multiple assays in research and in diagnostics, which is highlighted by several recent publications using NGS methods for large-scale SARS-CoV-2 testing (*8*–*10*). NGS-based tests can meet the needs for orders-of-magnitude scale-up and also provide ubiquitous genotyping data. Current NGS methods are reagent intensive and follow a laborious multistep protocol before sequencing. We developed ApharSeq, which is an early pooling protocol that markedly streamlines the NGS workflow and reduces reagent and labor costs. We established key properties of our approach, namely, linearity, sensitivity, low cross-reactivity, and the potential for multi-target testing. We also demonstrate that ApharSeq can provide high-confidence variant calling and even detect minor sample variants because of the UMIs introduced in the first step of the protocol. These variants are crucial for a more complete understanding of the evolution process the virus is undergoing, and might help in detecting infection chains in the wild (*33*, *38*, *39*).

Multiple differences exist between recently published NGS-based diagnostic assays (*8*–*16*), including sample type (e.g., saliva and nasal swab) and reaction type [e.g., RT-PCR and loop mediated isothermal amplification (LAMP)]. Early hybridization and pooling as implemented in ApharSeq are compatible with various extraction methods, enzymatic reactions, and protocols. A concern in early pooling assays, such as ApharSeq, is the competition between pooled samples during amplification, resulting in a markedly uneven coverage of samples in the same pool, potentially reducing sensitivity and increasing sequencing costs. However, we find that ApharSeq can detect a positive sample across a large dynamic range within the same pool, with relatively shallow sequencing. Furthermore, positive controls provide an intrinsic measure for sequencing depth in each pool, allowing for deeper sequencing where needed. ApharSeq incorporates UMIs into the protocol, providing several benefits: (i) The protocol is approximately as quantitative as qPCR across a 10^5^ dynamic range (Fig. 6B), (ii) the protocol provides higher confidence in genotyping even with a small number of observed molecules (Fig. 7), and (iii) erroneously assigned reads due to barcode hoping can be filtered out (see supplementary note on barcode hopping). For future development, different features implemented in various NGS approaches can be incorporated into a single protocol. These include the introduction of synthetic internal controls in each sample (*8*, *9*), testing for comorbid or confounding pathogens [e.g., the flu (*8*–*10*)], amplifying viral variable regions to investigate infection chains (*40*, *41*), and monitoring the host immune response with key transcripts (*42*).

NGS-based methods have notable limitations. They require specialized and expensive sequencing equipment, which has a relatively slow readout process (e.g., a 55-bp run on a NextSeq requires ~7 hours). In addition, NGS methods incur substantial overhead costs due to the fixed cost of a sequencing run regardless of the number of samples tested and are therefore unfavorable in low-throughput settings. However, a rough calculation shows that equipment costs per unit throughput are in favor of sequencing (see the Supplementary Materials), and when amortizing over multiple samples, sequencing reagent costs are reasonable, ranging from $10 per sample on a MiSeq (100 samples) to well below $1 per sample (thousands of samples on a NextSeq/NovaSeq). Regarding the end-to-end assay duration, a 12- to 24-hour turnaround time is not suited for emergency testing but can be useful for large-scale and routine population testing as it represents a reasonable trade-off with costs and labor reduction. Further optimizations and workarounds can reduce sequencing runtimes by a factor of 2 to 3 (*43*), potentially reducing turnaround to under 12 hours.

Each ApharSeq sample requires ~25,000 reads (see supplementary note on sequencing requirements), which means that a single Illumina NextSeq run with 400 million reads is sufficient to process 16,000 samples. The barcode design that we present here is amenable to changes, depending on the final sequencing scheme and expected throughput, but it is possible to design sufficiently distant 192 RT barcodes (pairwise edit distance >3) and 1536 pool barcodes (pairwise edit distance >2) to allow for the sequencing of ~300,000 samples in a single run. A linear increase in read length will result in a multiplicative scale-up of these numbers, effectively providing limitless pooling capability with an appropriately designed protocol.

Although increasing the size of the initial pool is beneficial in terms of labor and cost, we did encounter a slight inhibitory effect when pooling ~100 samples, suggesting that other unknown factors might limit the final pooling strategy. Last, we note that the approach developed and validated here, hybridization of barcoded primers followed by early sample pooling, is a generic protocol that can potentially be used to enhance existing protocols, including single-cell and bulk RNA-seq protocols.

## MATERIALS AND METHODS

### Study design

The goal of the clinical test was to find the extent of quantitative agreement between a benchmark qPCR assay and ApharSeq and allow us to estimate the LoD for our assay. Clinical samples were collected in the Clinical Virology Laboratory at Hadassah hospital. This study was part of the approved diagnosis optimization and validation procedures at the Hadassah Medical Center, and therefore, no additional Institutional Review Board approvals were required.

Around 900 negative and ~100 positive samples were obtained. The only information that we had was that the samples were positive or negative. We randomly assigned positive samples to plates to have an average positivity rate of 7.5% per plate (.i.e., final ApharSeq pool). As a negative control, we randomly assigned at least six wells in each plate to contain only water, and as a positive control, we diluted a positive sample in a pool of negative samples so that we had sufficient volume to allocate two aliquots of an identical positive control to each plate (in constant well positions). Unnamed negative samples were distributed sequentially to fill the remaining positions in each plate. Each plate was split and subjected to qPCR with a standard FDA-approved kit (BGI) and ApharSeq.

In 4 of the 10 plates, positivity rate exceeded 50% by qPCR, and together with the clinical laboratory staff, we concluded that the negative samples in these plates were likely contaminated in the laboratory. These four plates were discarded from further analysis. ApharSeq pools were subjected to an automated script on an EVOware 100 Tecan system, and the pools were stored in RNAlater for further processing following the ApharSeq protocol (detailed below).

### RNA extraction benchmarking

Viral RNA at Ct ~14 was extracted from an in vitro grown virus (SARS-CoV-2 isolate USA-WA1/2020, NR-52281; obtained from BEI Resources) and serially diluted 1:25 in a negative sample. Each dilution was subjected to RNA extraction using one of three methods: (i) 400 μl of sample with Quick-RNA MagBead (Zymo Research), following the kit manufacturer’s instructions; (ii) 400 μl of sample following the polyT capture described below; and (iii) SPRI-based capture as published (*21*) with modified volumes: 152 μl of sample, 51 μl of beads, 153 μl of polyethylene glycol (PEG) buffer, and 122 μl of binding buffer. Samples in the linear range were corrected for their dilution and collated to estimate the mean relative yield and error for each extraction method (Fig. 2A).

### Primer design

All oligos used in this study are provided in a supplementary excel spreadsheet, and a detailed description with examples is available in the Supplementary Materials.

#### RT primers

RT primers consist of four main parts—from 5′ to 3′—a general Illumina handle (Nextera R1), a 10-bp UMI, a 10-bp barcode, and a target-specific primer. After the first iteration of sequencing experiments, we decided to (i) interleave the UMI (U) and barcode (B) to avoid long stretches of the same nucleotide in the UMI sequence and (ii) add to each primer a variable sequence of 0 to 2 N’s before the amplicon primer to increase complexity in each sequencing cycle.

#### PCR primers

There are two different PCR strategies that we used during the development process—one-step and two-step PCR. The two-step PCR is composed of a first step that amplifies the target molecules with an extendable handle, and in the second step, barcode and the remaining Illumina sequences are introduced. The one-step reaction performs everything in a single reaction with a single long primer (~90 bp). A one-step reaction is more convenient and is less prone to contaminations (see supplementary note on contaminations) but is less modular. The long primer contains a target-specific sequence and a barcode, which means that a barcoded primer collection must be synthesized per target. The two-step PCR decouples this dependency, which means that a single collection of barcoded primers can be used on any target, assuming that a simple target-specific primer is used in the first step. Both approaches yielded similar results, and we are currently using the one-step reaction to avoid contamination.

#### One-step PCR

The PCR amplifies the generic handle on the RT primer on one side and a target-specific sequence on the other side. In addition, the PCR extends the amplicons to a sequencing library by adding the relevant flanking Illumina sequences. An 8-bp barcode was included that marks the pool of samples amplified in the PCR.

#### Two-step PCR

The first step adds a target-specific handle on the forward side and extends the generic handle in the reverse (RT) side of the amplicon. We do this with the published Tn5-Rd1/Rd2 (Illumina FC-121-1030). The second PCR step extends the handles to a complete library with the Ad1.x and Ad2.x indexed primers as published (*44*).

### ApharSeq protocol

The detailed and complete protocol was published separately (*45*). A specific instantiation of the ApharSeq protocol using polyT beads, assuming the use of 96 barcoded RT primers and 96 barcoded PCR primers, is given in the Supplementary Materials. Detailed steps are described below. Because the protocol stabilized with time, some experiments were slightly modified relative to the current protocol. The Supplementary Materials also contain a list of experimental modifications per experiment shown indexed by figure panel.

### Bead preparation

We tested commercially available polyT beads (Thermo Fisher Scientific dynabeads, catalog no. 61002), or conjugated carboxylate-coated beads (GE Healthcare Sera-Mag SpeedBeads, catalog no. 65152105050250), and followed the manufacturer conjugation protocol with a 25-dT oligo.

### Hybridization and RNA purification

#### Option 1: Purification and hybridization on polyT beads

This RNA purification protocol is based on a protocol for rapid isolation of mRNA (*46*) with some modifications. Briefly, polyT-conjugated beads were washed once and resuspended in binding buffer. The resuspended beads were mixed 1:1 with the sample. After a hybridization period of 10 min at room temperature with periodic mixing, the supernatant is removed and the beads are resuspended in a 50-μl 1:1 mix of binding buffer and 10 μM barcoded RT primers. To denature RNA secondary structures, the samples were incubated at 72°C for 2 min and immediately transferred to ice for at least 2 min. Samples were then incubated at room temperature for 10 min with periodic mixing to allow hybridization of RNA to the beads and to RT primers. Beads were resuspended in 450 μl of wash buffer A and magnetized. The majority (380 μl) of the supernatant was removed, and beads were resuspended in the remaining 70 μl of buffer A and pooled. After pooling, samples are washed once in buffer A and twice in buffer B and can be kept in RNAlater until they are processed further. Preliminary tests show that RNA can be stored on the beads in RNAlater at 4°C for at least a week.

#### Option 2: Purification and hybridization on SPRI beads

RNA extraction with SPRI beads followed our published protocol for RNA extraction (*21*, *46*) with several modifications. Samples in lysis/transfer buffer were mixed with barcoded RT primers, then incubated at 72°C for 2 min, and immediately transferred to ice for at least 2 min. Samples were then mixed 1:1 with binding buffer (as above) and incubated at room temperature for 10 min with periodic mixing to allow primer hybridization. Next, samples were mixed 1:0.8 with homemade SPRI beads in PEG buffer. Beads were washed twice with freshly made ethanol (80%), air-dried, and eluted in double distilled water. This was followed by a second 0.8× SPRI cleanup to ensure the removal of any excess primers. At this stage, samples were pooled to a PCR tube to undergo RT and PCR.

### cDNA synthesis and library preparation

Twenty-five percent of pooled beads were subjected to proteinase K treatment (Lucigen), washed, and underwent RT reaction with SMARTScribe enzyme (SMARTScribe Reverse Transcriptase, Takara Bio) at 42°C for 1 hour followed by incubation at 70°C for 15 min. To elute the cDNA from the beads, the samples were incubated at 98°C for 2 min and magnetized, and the supernatant was transferred to a new tube and cleaned by SPRI beads x2 (Agencourt AMPure XP, Beckman Coulter). Illumina adaptors were added by PCR (30 cycles; KAPA HiFi HotStart ReadyMix, Kapa Biosystems), and the DNA was purified using 1× SPRI.

### NGS data analysis

Reads were demultiplexed using bcl2fastq (version 2.20.0) and further processed by ad hoc Python scripts that are available as Jupyter Notebooks (10.5281/zenodo.5069979). For UMI analysis, we found the “uniq+” measure to be a simple and useful heuristic—we collapse unique UMIs and only count those with more than one associated read. This strategy might result in some undercounting in undersequenced samples, but this is a less important issue than counting spurious UMIs due to physical contamination, barcode hopping, or sequencing errors. A more detailed discussion is given in a supplementary note on UMI analysis.

### Quantifying target molecules

To generate a quantitative polyA viral reference, we extracted RNA from a clinical sample and estimated the number of molecules in this RNA extract to be 6000 molecules/μl using a synthetic viral sequence (Twist Bioscience SARS-CoV-2 RNA, MN908947.3) as a reference in a standard RT-PCR kit. We loaded two samples with 10 and 5 μl (~60,000/30,000 molecules) of this reference RNA in a total of 320 μl of lysis buffer and applied the ApharSeq protocol. Only ^1^/_30_ of the pooled material underwent library preparation. Therefore, we expect to see ~2000 or ~1000 molecules in the 10- or 5-μl samples, respectively. After UMI clustering, we observe 33 and 14 molecules, respectively, suggesting that we capture ~1.5% of molecules. In the same experiment, a sample corresponding to cycle 29.3 had a similar UMI count (32 molecules), allowing us to roughly calibrate the Ct units to target molecules per milliliter at Ct 29.3 = 6150 × 10/0.32 = ±190,000 molecules/ml.

### LoD determination

#### Titration LoD

For the high load titration (Fig. 4B), a linear fit (python scipy.stats.linregress) was performed

y~b+ax

where *y* is the log_10_(#UMIs) and *x* is the calculated Ct of the sample. Given this linear fit, we can extrapolate to the UMI detection threshold, which, in this case, was set to 3 (a conservative estimate). The fit statistics are

**Table:** 

**Statistic**	**Singles**	**Pooled**
Slope (*a*)	−0.242	−0.276
Intercept (*b*)	9.55	10.3
*P* value	2.86 × 10^−4^	2.53 × 10^−4^
*R*^2^	0.99	0.99
Ct @ log_10_(3)	37.48	35.7

For the low load titration (Fig. 4, C and D), we perform resampling of the data (×500 times):

For each factor in (1, 3, 10, 30, 100, 300, 1000)

For each sample

For each UMI in sample

#sampled-reads(UMI) ← Poisson(#reads(UMI)/factor)

We then count the number of UMIs per (sample, factor, replicate) as the number of UMIs with #sampled-reads(UMI) > 0. Given these counts, we set the detection threshold as the minimal number of UMIs that is above 99% of replicates in the negative samples. Therefore, this number varies with sequencing depth and the UMI background in the negative samples. We fit each sampled replicate of the data with a Poisson-noised exponent

$$y\sim \text{Poisson}(2^{b-\mathrm{ax}})$$

where *y* is the number of observed UMIs and *x* is the calculated Ct of the sample. We then set the LoD per factor to be the maximal Ct such that 95% of replicates are above the LoD.

**Table:** 

**Resampling** **factor**	**Average no. of** **reads** **per sample**	**UMI** **threshold**	**LoD (Ct)**
×1	247,700	3	38.0
×3	82,600	3	37.8
×10	24,700	3	37.4
×30	8,260	3	37.2
×100	2,470	3	36.9
×300	826	2	37.2
×1000	248	2	36.8

#### Clinical test LoD

Ct 35 was used as a cutoff value for positive/negative samples, as is currently used in approved diagnostic protocols. We first determine the false discovery rate (FDR) threshold by fitting a zero-inflated Poisson mixture model (two components + zero component) to the molecule counts observed in negative samples. Using this model, we determine the theoretical threshold of detection for a given FDR. In this case, when FDR is set to 5%, the threshold is five molecules.

We fit the positive samples with a linear model (slope = −1), assuming Poisson noise. Given the linear model, the FDR threshold (five molecules), and assuming Poisson noise, the LoD is determined to be the maximal Ct in which the probability of obtaining a value lower than the threshold is lower than 5%. We subsample reads from the data and repeat this analysis to every sequencing depth to obtain the LoD as a function of sequencing depth (Fig. 6C).

### Viral sequence variation analysis

As a proof of concept, we designed primers spanning the P681H mutation and the N501Y and E484K mutations, allowing us to distinguish between the alpha, beta, and gamma variants. We first cluster reads by their UMIs and then count the observed viral sequences associated with each UMI. If a UMI was observed more than two times and >50% of reads associated with that UMI have the same observed viral sequence, we call that sequence the major sequence for that UMI. There are two cases in which the major UMI sequence will have a mutation relative to the reference (or consensus) sequence: (i) An RT error had occurred and (ii) the original RNA molecule had a mutation.

The first case occurs once every ~30,000 reverse-transcribed bases (*47*), i.e., the probability of a single base to have a specific error (e.g., G to A) is roughly 1:90,000, which will be the (theoretic) false detection rate of a specific mutation.

To estimate the LoD, we use the samples that were tested with the P681 amplicon. We have four samples

**Table:** 

**Genotype**	**Ct**	**No. of unique** **molecules**
P681H	20.7	32,984
P681H	20.0	25,641
P681H	22.0	15,120
P681P	20.5	21,653

Extrapolating from these numbers, we predict that five molecules should still be observable at Ct 31 with >95% confidence.

## Supplementary Materials

### This PDF file includes:

Materials and Methods Figs. S1 to S7 Tables S1 to S3 References (*50*, *51*)

## Notes

View/request a protocol for this paper from *Bio-protocol*.
