# Supplementary material for: Early sample tagging and pooling enables simultaneous SARS-CoV-2 detection and variant sequencing
Source: Sci Transl Med. 2021 Nov 3;13(618):eabj2266. doi: 10.1126/scitranslmed.abj2266 (PMC9928115; doi:10.1126/scitranslmed.abj2266)
Supplement: Supplementary file 1 — Materials and Methods Figs. S1 to S7 Tables S1 to S3 References (50, 51) [file scitranslmed.abj2266_sm.pdf]

Science Translational Medicine
AAAS

Supplementary Materials for

Early sample tagging and pooling enables simultaneous SARS-CoV-2 detection and variant sequencing

Alon Chappleboim *et al.*

Corresponding author: Nir Friedman, nir.friedman@mail.huji.ac.il

Sci. Transl. Med. **13**, eabj2266 (2021)
DOI: 10.1126/scitranslmed.abj2266

The PDF file includes:

Materials and Methods
Figs. S1 to S7
Tables S1 to S3
References (*50, 51*)

Other Supplementary Material for this manuscript includes the following:

Data file S1

Supplementary Materials and Methods

UMI Analysis

In an experiment like one depicted in Figure S5 (same sample with a mix of odd/even column primers), we performed a UMI bias analysis. The following figure compresses the UMI position weight matrices (PWM) per primer. This is an example of a PWM for the ActB primer at well A1:

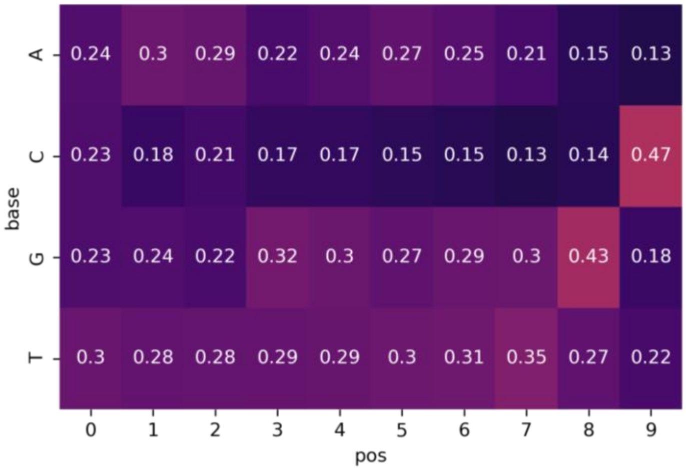

PWM for ActB primer at A1 well. numbers and colors indicate the relative abundance of each base in each position along the UMI sequence (0-9).

An extension of the above figure to the entire plate:

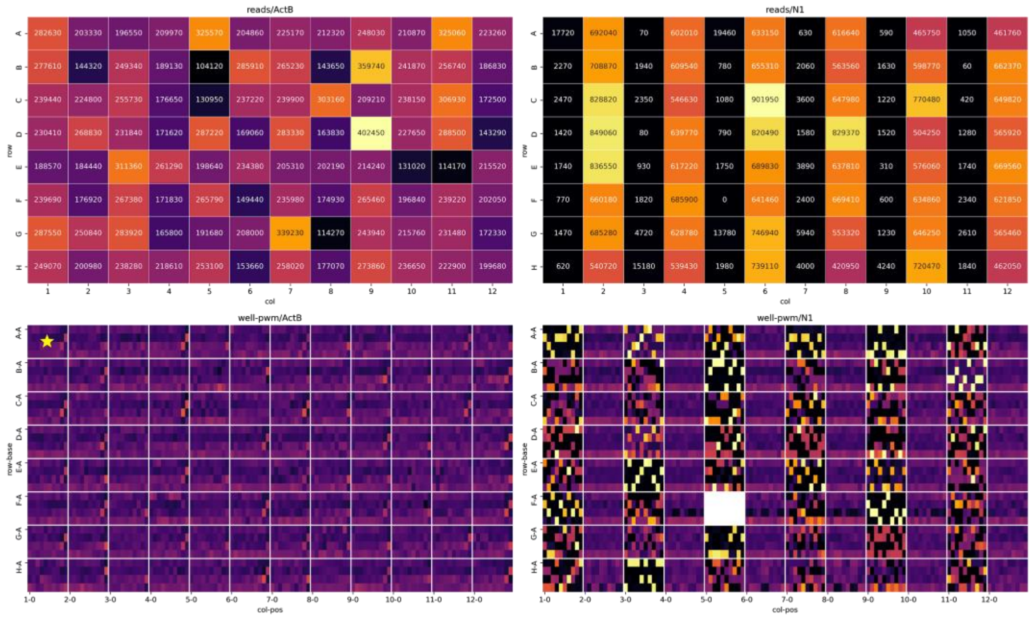

PWM for all the RT primers. Top row shows the number of reads in each position in this specific experiment. Bottom row is an extension of the previous figure (yellow star indicates the corresponding submatrix to the previous figure).

The distribution is very close to a uniform distribution, with a notable exception in the last two positions, and as indicated by the following figure, which varies by the offset that separates the UMI from the primer.

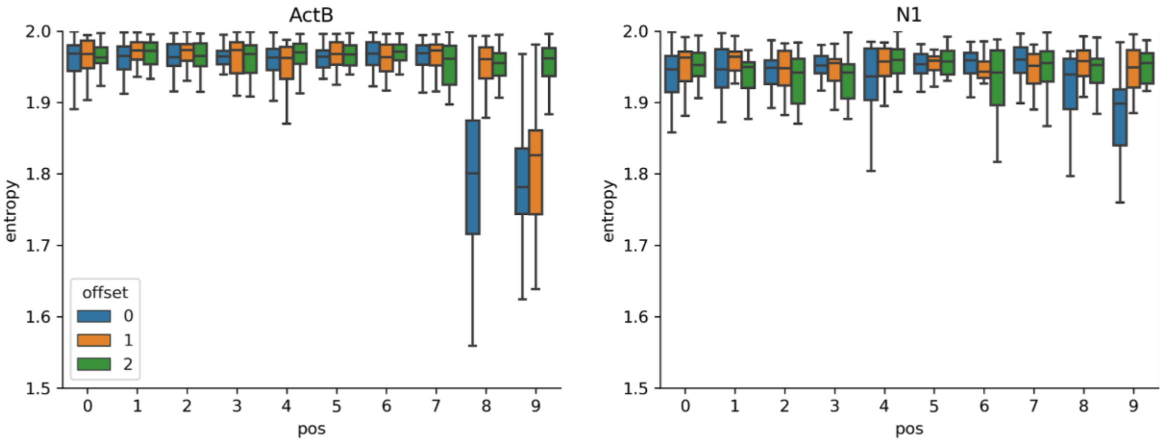

UMI position entropy as a function of primer and position. N1 analysis is limited to the odd columns. Note that entropy is very close to the theoretical 2bit of the uniform distribution.

A 2-base offset seems to be sufficient to mitigate this phenomenon, and in future primer sets, the offset should vary between 2-4.

Barcode Hopping

To test barcode hopping in the above experiment we looked at the read counts in cross-barcode combinations in unexpected combinations:

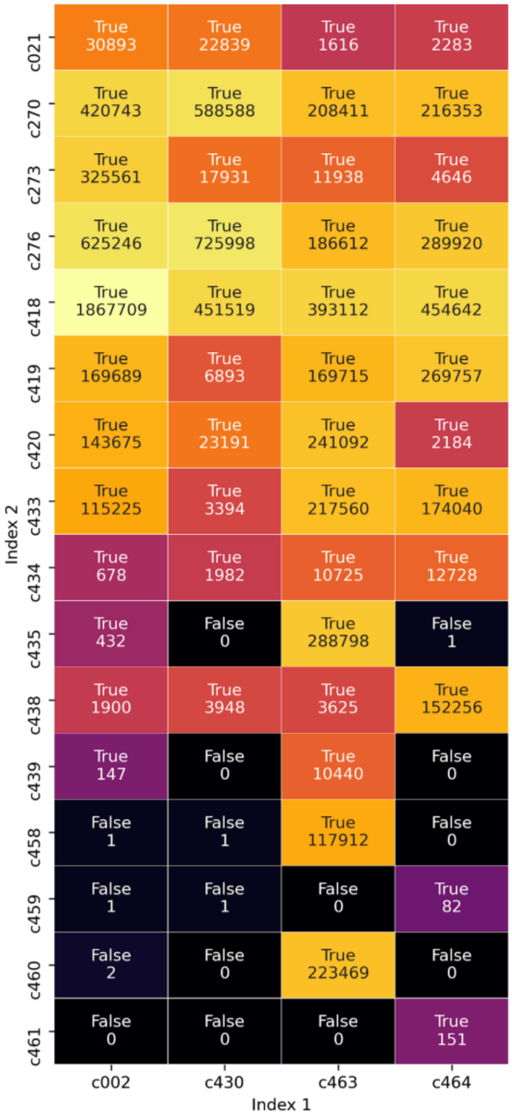

Little to no barcode hopping. A heatmap showing the number of reads per combination of index1 and index2, and an indication whether that combination was expected (“True”) or not (“False”).

There is evidence of very little barcode hopping. For example, the combination of c002 and c458 has only a single associated read, despite the fact that there are ~120k reads with barcode c458 and ~3.7M reads with

barcode c002. Assume 1 in 100k reads hops between libraries, the probability of more than one read with the same UMI hopping between libraries is extremely small. Filtering UMIs with more than one supporting read makes barcode hopping negligible. Alternatively, if this filter is too restrictive, a slightly more sophisticated approach, whereby a probabilistic model assigns UMIs to their most likely sample of origin is straight-forward, albeit more computationally intensive.

Sequencing requirements

The sequencing requirement for viral detection using ApharSeq is reading a single pool barcode (8-12 bp) and at least 50 bases from R1, or 20 bp from R1 (which covers the sample barcode and UMI) and 30 bp from R2. When we down-sampled the data in the titration experiment, we found that a change of x1000 in sequencing depth only incurs a 2-fold reduction in sensitivity (Fig 4D). Additionally, using the Ct distribution observed in the clinic, we performed a simulation to estimate the false positive and false negative rate as a function of sequencing depth (Fig. S5). In these simulations, when sequencing depth ranged from 10,000 to 100,000 reads per sample, the false negative rate ranged from 4.5% to 0.2% respectively. We conclude that 50,000 reads per sample on average should suffice. A parallel analysis was done that down sampled the clinical test data and showed that 25,000 reads per sample are sufficient (Fig 6C). This means that a single Illumina NextSeq 500/550 run with 400 million reads suffices for processing 8,000-16,000 samples, and a NovaSeq S2 100bp run with 8 billion reads allows the processing of 160,000-320,000 samples simultaneously.

RT primer performance variation

After experiencing some issues with a batch of barcoded primers we decided to test the primers for variation and contaminations. We pooled 96 primers into a single pool twice, and hybridized them, as a pool, to a single positive and single negative sample. If any of the primers is contaminated, a library should arise from the negative sample and then a search for the contamination should ensue. Similarly, any variation in the number of UMIs observed indicates a barcode-specific issue, probably related to synthesis or a step prior to the RT reaction. Another test for primer variation can examine differences in the distribution of reads/UMI, suggesting PCR amplification biases, but since UMI-counting should mitigate such differences, PCR biases are a lesser concern.

When we perform this test (Figure S4) , we see most variation in UMI counts is primer-specific. We believe that this is mostly due to synthesis differences. More than >80% of primers fall within a ±25% range around the mean, indicating that while this issue should be addressed, it is not detrimental to the implementation of ApharSeq on a large scale.

Contamination issues and best practices

While working on the development of the assay, we encountered significant PCR contamination issues. Specifically, previous sequencing-ready libraries are a potential contaminant and are re-amplified in subsequent PCR reactions. Once we realized this was an issue, we could detect these contaminants experimentally by including negative controls in the library PCR reactions, and computationally by the checking for the existence of reads with RT primers that were not used in the specific experiment, and by comparing the UMI pools of previous and current libraries in cases where they shared the same RT primers. For the time being this issue seems to be under control in our lab. To achieve this, we separated the work space to pre-PCR and post-PCR. No reagent or device is used in both spaces, and there are dedicated lab coats to each space. Any processing performed on the final library - quantification by qbit, tape station, etc. - is done only in the post-PCR space. See Aslanzadeh for more information *(50)*.

Additionally, we observed contamination between primers in the synthesized oligo plate we received from the manufacturer. We highly recommend testing each plate as it arrives for primer contamination and uniformity (see Figure S4, S6 above). This issue seemed to have been resolved when we ordered the oligos in dry form and dissolved them in the lab.

Required equipment and plastic consumables

Preparation and consumables per plate:

- 25 µl 10 µM barcoded primers are distributed with in 25 µl binding buffer to a 96 PCR plate and stored in -20°
- 500 µl wash A is distributed to a 2 ml deep-well plate
- Samples are ordered in a 2 ml deep-well plate by a dedicated robotic system
- A single 96-well 150 µl filter tipbox per plate is used (Tecan)

RNA cleanup and hybridization

Performed by the MCA on the robotic system using a single tip box in ~40 minutes and ends with a single tube containing the pool of the 96 samples in a single well of the plate in RNA*later* for long-term storage.

Our robotic setup includes an Evo robotics system by Tecan with:

- Deep well magnet
- Deep well shaker (BioShake 30125516)
- Deep well heating block (BioShake 30165954)
- Deep well cooling block (BioShake 30165954)
- 96 MCA arm
- 8 channel LiHa arm

Enzymatic reactions

Pools are reordered in a 96 plate and processed with a multichannel / robotic liquid handling.

Required equipment (we use standard 96 equipment, but eppendorfs or PCR strips can also be used for lower throughputs):

- 96 well plate PCR block
- 96 well plate shaker
- 96 well plate magnet

Sequencing

We quantify resulting DNA libraries with the Qubit (ThermoFisher) and TapeStation (Agilent), and sequence on a NextSeq 550 (Illumina).

In terms of infrastructure costs, the main device uniquely required for ApharSeq is a sequencing machine. RT-PCR machines range from $15,000-90,000 per machine *(51)* , while a single NovaSeq machine costs ~$900,000. Thus, at worst, a single NovaSeq machine is equivalent to ~60 RT-PCR machines, and at best, these machine process 384 samples in ~90 minutes. A single NovaSeq run will take ~7.5 hours, which is ~5 runs of an RT-PCR machine, ~ 60x5x384 qPCR samples = 115,200 samples, which equals 35-70% of the NovaSeq estimated throughput (160k-320k samples per run).

A note on RNA capture methods

Since we aimed for an RNA-seq based detection assay, we first needed to extract RNA from the lysed clinical samples with standard, high-throughput nucleic acid cleanup techniques. We tried three different approaches: polyT paramagnetic microbeads (commercial, and home-made variants), paramagnetic microbeads conjugated with viral-specific bait oligos, and SPRI beads for general nucleic acid cleanup.

The viral-specific beads yielded poor results, and we discontinued this experimental approach. The polyT/SPRI branches gave high viral RNA yields, comparable to common RNA extraction procedures (Figure 1A), and were compatible with downstream standard RT-qPCR kits. We performed extensive tests on SPRI extraction (Figure S1) and performed several library preparation tests based on the SPRI cleanup

(Figure S1). These tests demonstrate that it is a viable alternative to the homemade polyT beads we are currently using, and that with further optimizations might even provide higher sensitivity (Figure 1A). Both techniques are based on the Sera-Mag SpeedBeads modified with a carboxylate residue by GE healthcare (Cat# 65152105050250) Both variants allow large scale batch preparation and long-term storage and usage. See Rahat et al. *(21)* for more details on the SPRI protocol, and the "Bead conjugation" and "Hybridization and RNA purification" sections in the methods part of this manuscript.

FNR/FPR estimation by sampling

The Ct distribution was obtained from the Clinical Virology Unit of Hadassah Ein Kerem Hospital. To estimate the false negative/positive rates we perform the following sampling procedure:

Given:

- A pool size (e.g. 96 samples)
- The population-wide positive sample rate (e.g. 5%, a high estimate)
- Background (0.25 was used, i.e. ¼ read on average per negative sample), and
- The positive Ct distribution

We sample the number of molecules per sample and assume that Ct 26 is ~11,000 molecules per assay (~35,000/ml *(31)*). A UMI is sampled for each molecule (assuming uniform synthesis), and sequencing errors are introduced. The molecules are pooled, and the total number of reads assigned to the pool (e.g. 96,000 reads in the case of 1,000 reads per sample) is sampled from that pool, with replacement (accounting for PCR amplification). UMIs are collapsed, and unique molecules are counted. The detection threshold was set to arrive at a false positive rate of 1 in 1000 (assuming the background is correct), and the false negative rate is empirically determined by the number of samples that are positive but have less observed molecules than the detection threshold.

ApharSeq instantiation

The following is a specific instance of the protocol:

Per sample (pre ApharSeq)

1. Collect an NP swab sample
2. Remove 225 µl NP swab sample and mix with 175 µl Lysis buffer (Zymo RNA/DNA shield x2)
3. Place 320 µl of mixed sample in a 96 deep well plate

Per sample (~40 minutes, 96 samples in a 96 sample plate, currently executed robotically)

4. Add binding buffer and hybridize to polyT magnetic beads for 10 minutes
5. Magnetize and remove supernatant
6. Add primers and hybridize to RT primers:
    a. Heat to 72° for 2 minutes
    b. Cool to 4° for 2 minutes
    c. Incubate at 25° for 10 minutes
7. Magnetize and remove supernatant
8. Resuspend beads in wash buffer, and pool samples to a single tube
9. Wash and resuspend in RNA-preserving buffer

Per pool (~240 minutes, ~9200 samples in a 96 sample plate)

10. Resuspend beads in proteinase K reaction buffer and incubate for 60 minutes at 37°
11. Wash and resuspend beads in reverse transcription buffer and incubate for 60 minutes at 42°
12. Elute cDNA from beads by heating to 98° and removing the sup
13. SPRI x2
14. Resuspend cDNA in PCR reaction mix
15. Perform PCR
16. Pool 2 µl of each pool

Per pool of pools (~600 minutes, ~880,000 samples in a 96 sample plate)

17. SPRI x1
18. Quantify DNA, dilute to 4nM
19. Load sequencing machine to have ~25k reads per sample

    NextSeq - upto 16,000 samples, or roughly 2 pools of pools

    NovaSeq - upto 320,000 samples, or roughly 40 pools of pools

Analysis

20. Load sequencing machine to have ~25k reads per sample
21. Count only reads that conform to the expected format:
    a. Exact match to pool barcode
    b. Exact match to RT barcode
    c. Exact match to first 8 bases of the RT primer
    d. Exact match to first 8 bases of the target amplicon (N1/ActB)
22. Count reads per sample/target/UMI, and consider combinations with at least 2 reads as observed molecules
23. If the sample has more than 5 observed N1 molecules

    Then the sample is considered **positive**

    Else, if the sample has less than 4 observed ActB molecules

    Then the sample is considered a **failed** test and need to be repeated

    Else, the sample is considered a **negative**.
24. For positive samples, collate per-base information from vetted UMIs (>2 reads with >50% agreement on base identity) to determine minor and major variants per sample per observed genomic position

Primer Design

RT primers

From 5' to 3':

- Nextera R1 sequence handle - **GCGTCAGATGTGTATAAGAGACAG**
- UMI/Barcode - **UUUUBBBBBUUUUBBBBBUU**
- Offset - **N{0,2}**
- Target primer -
    - N1 **tctggttactgccagttgaatctg**
    - E **atattgcagcagtacgcacaca**
    - ActB **acagcctggatagcaacg**
    - P681 **ctacactacgtgcccgccg**
    - N501 **ctctgtatggttggtaaccaac**

  Example of a specific primer (c410 - ActB RT primer):
  GCGTCAGATGTGTATAAGAGACAGNNNNACCTCNNNNTATCANNNacagcctggatagcaacg

One-step PCR primers

Reverse primer (generic):
CAAGCAGAAGACGGCATACGAGATGTGACTGGAGTTCAGACGTGTGCTCTTCCGATCT

Forward primer (target-specific, and indexed)

N1 **gaccccaaaatcagcgaaa**

E **acaggtacgttaatagttaatagcgt**

ActB **caccaactgggacgacat**

Example (c418 - ActB PCR primer):
CAAGCAGAAGACGGCATACGAGATTCGGACTAGTGACTGGAGTTCAGACGTGTGCTCTTCCGATC Tcaccaactgggacgacat

Two-step PCR reaction

1st reaction:

Reverse primer (generic):
TCGTCGGCAGCGTCAGATGTGTATAAGAGACAG

Forward primer example ("B6" - N1 PCR primer):
GTCTCGTGGGCTCGGAGATGTGTATAAGAGACAGgaccccaaaatcagcgaaa

2nd reaction (with standard Illumina sequences):

Reverse primer (example Ad2.1, barcode in red):
CAAGCAGAAGACGGCATACGAGATTCGCCTTAgtctcgtgggctcggagatgt

Forward primer (example, Ad1.1, barcode in red)
AATGATACGGCGACCACCGAGATCTACACCTCTCTATtcgtcggcagcgtcagatgtg

Supplementary Figures

Fig S1. RNA extraction with homemade SPRI beads

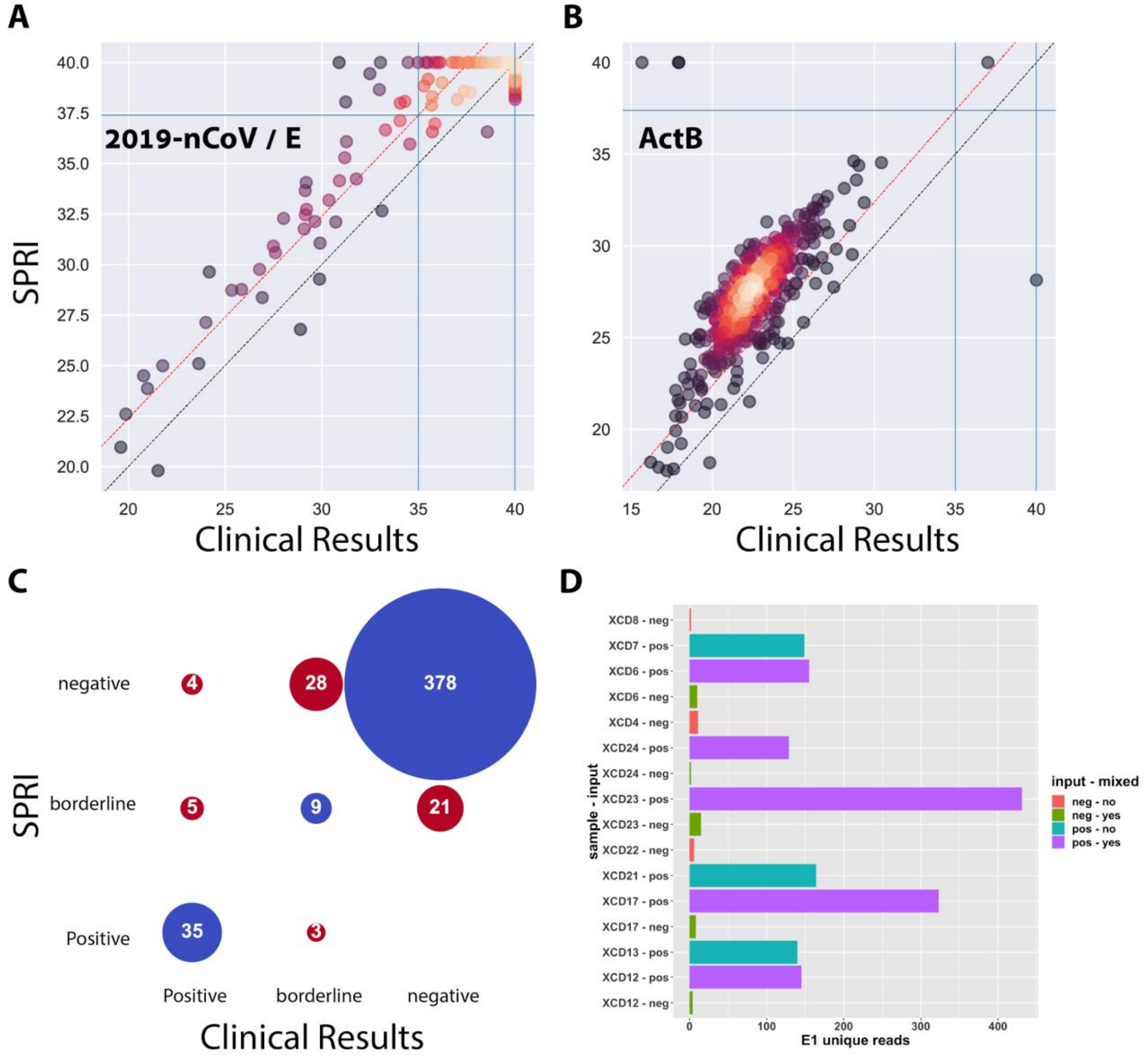

Supplementary Figure 1 - RNA extraction with home-made SPRI beads was published [1], and validated on hundreds of samples. Evaluation was performed by an approved PCR test kit [2]. Matched clinical results were extracted with standard kits (mostly Qiagene).

A) E gene Ct values as obtained from the clinic (x-axis) and as obtained after SPRI based extraction. Black dashed line indicates the x=y line. Red dashed line indicates the expected offset due to the difference in sample load to the qPCRs due to different elution volumes. Blue lines indicate the thresholds for determining the categories in **C**.

B) Human internal control Ct values in the same tests show high agreement between extraction methods overall.

C) Confusion Diagram comparing the clinical results vs. the SPRI extraction results. Blue circles are the cases in which there was an agreement (87% of total tests). Red circles show the cases in which there was disagreement between measurements.

D) SPRI-based extraction and Apharseq on the viral E amplicon. Purple/teal indicate positive samples when they were pooled/unpooled, respectively, green/red are matched negative samples that were pooled/unpooled respectively. Pooled negative samples have less unique molecules relative to their positive counterparts, indicating that cross contamination occurs to a minimal degree that can likely be optimized further.

Fig S2. Preliminary Optimizations

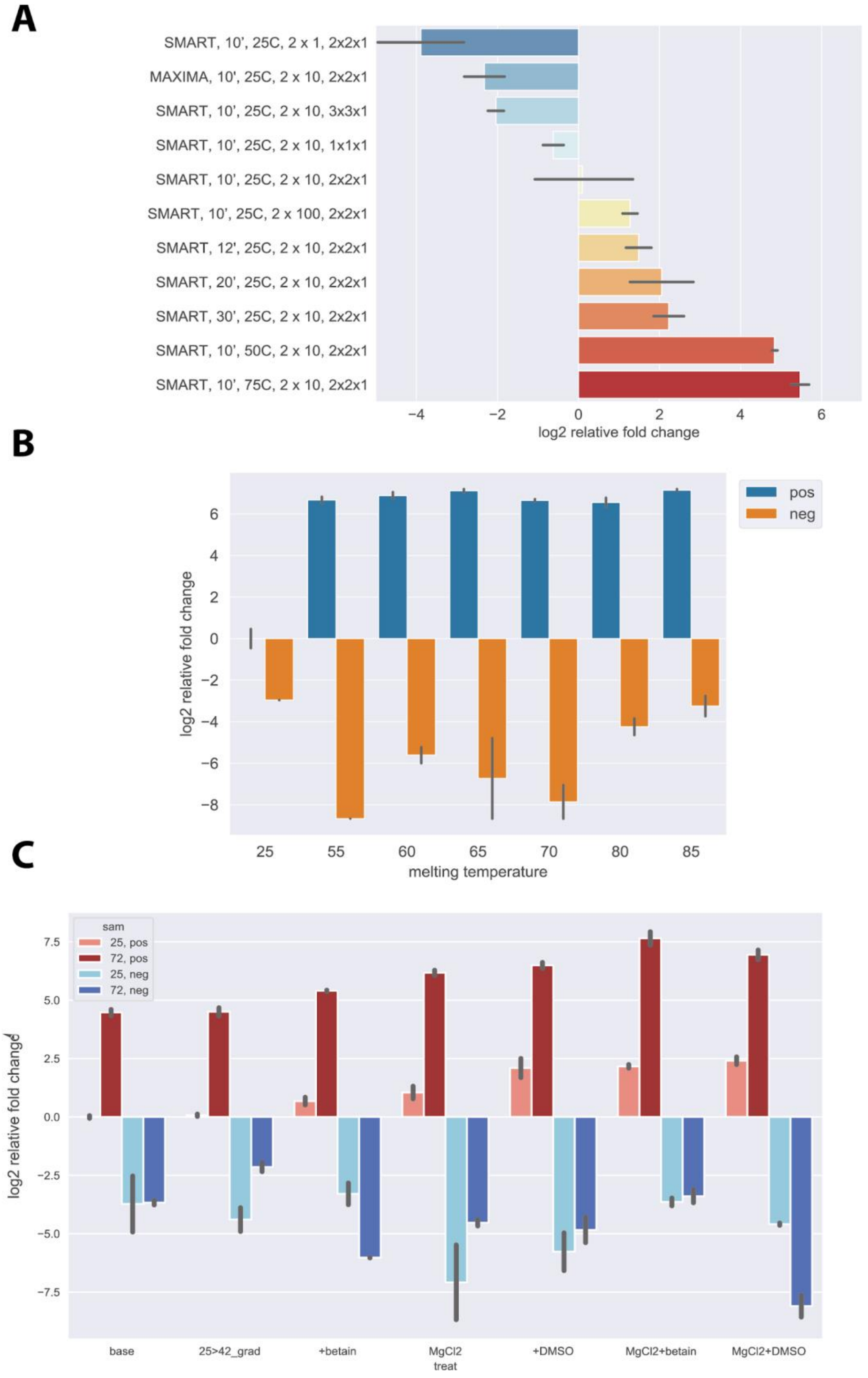

Supplementary Figure 2 - Preliminary Optimizations All tests were performed on the E amplicon using a primer-specific qPCR reaction (as shown in Figure 3A).

A) RNA hybridization/extraction. We tested different RT enzymes (Maxima/SMART), different hybridization durations (10’-30’), different temperatures prior to hybridization (25ºC, 50ºC, 72ºC), different RT primer concentrations (2 µl of 1/10/100 µM), and different wash regimes (AxBxRT).

B) RNA melting temperature. Sample preheating is crucial (relative to 25ºC), but any temperature above 55ºC appears to yield a similar amount of product.

C) RT reaction. Reaction conditions were tested relative to the “base” manufacturer’s conditions. Signal vs. background (dark red vs. dark blue) yield improved significantly in the $MgCl_2$ + DMSO condition.

Fig S3. Human Control Amplicon.

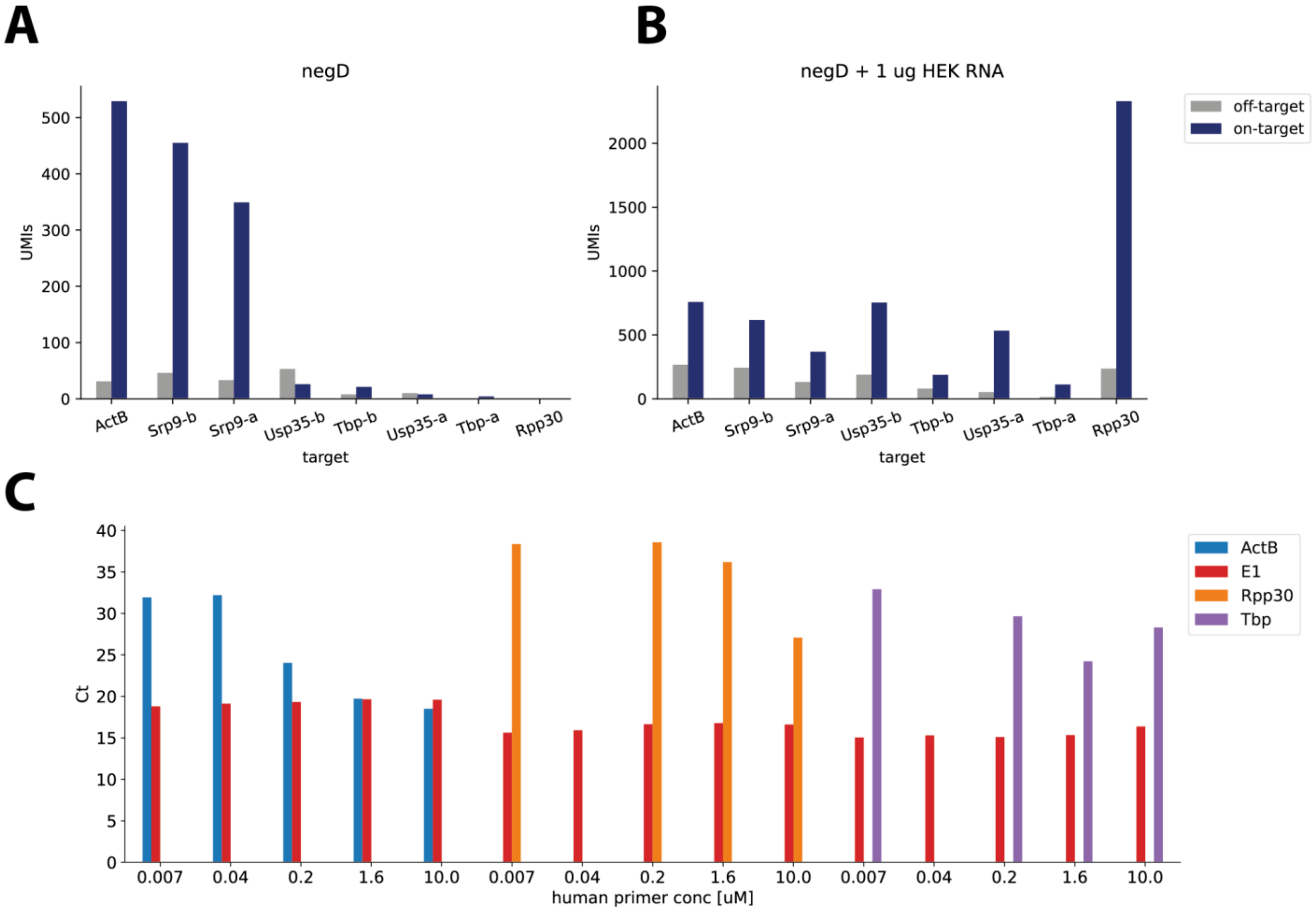

Supplementary Figure 3: Human Control Amplicon

A) All the targets tested on a pool of negative samples (extension of Figure 5C). “a”/”b” indicate different primer pairs on the same transcript

B) Addition of human RNA. Same primers used on the pool of negative samples supplemented with 1 µg of RNA extracted from HEK cells.

C) Attenuating relative amplicon abundance. The target-specific PCR primer concentration was varied in the library PCR to alter amplicon abundance. Assay is a qPCR test that is target+library specific. There is a dose-response relationship between primer abundance and product abundance (height of blue series, green series). Note that different amplicons appear to alter the E amplicon yield (height of orange bars)

Fig S4. RT barcode variations

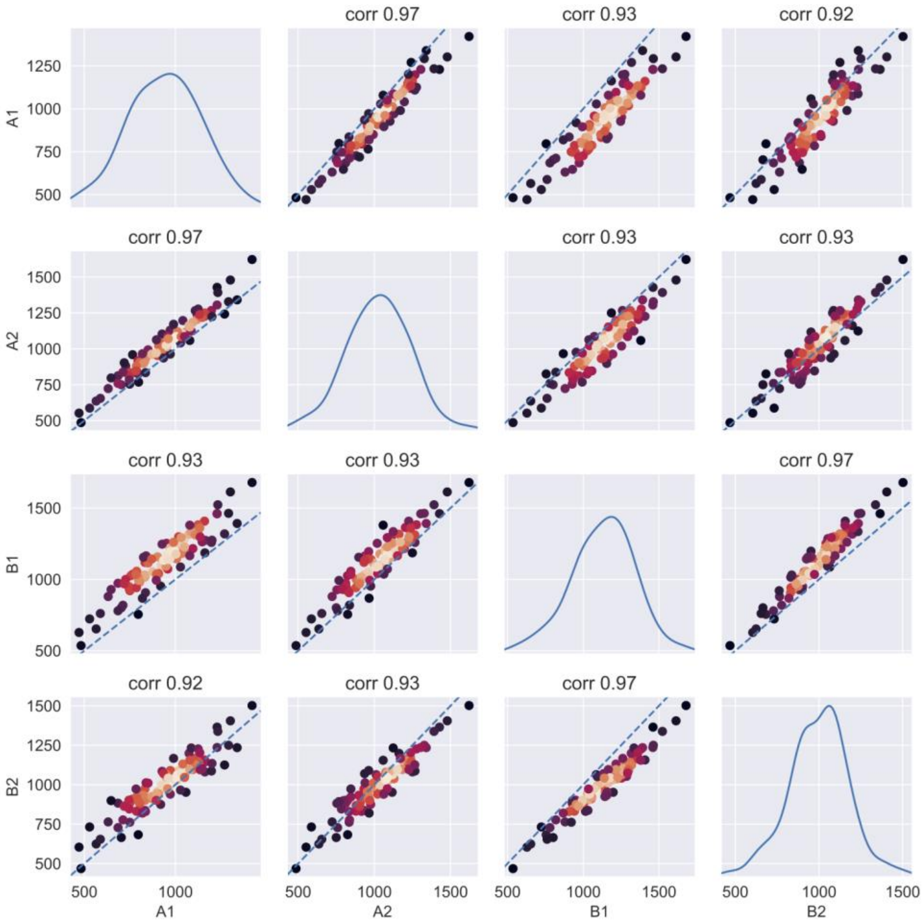

Supplementary Figure 4: RT barcode variations

We pooled 96 differently-barcoded RT primers twice (“A”/”B”) and hybridized these pools in two replicates to a positive sample (“1”/”2”). Plotted are the number of UMIs observed in each pool/replicate. Each dot is a specific primer. Primers are highly correlated between pools, suggesting that most variation is due to intrinsic properties of the primer or synthesis variation issues. In all replicates, >80% of barcodes are within a ±25% range around the mean.

Fig S5. Sequencing depth and FPR/FNR estimation

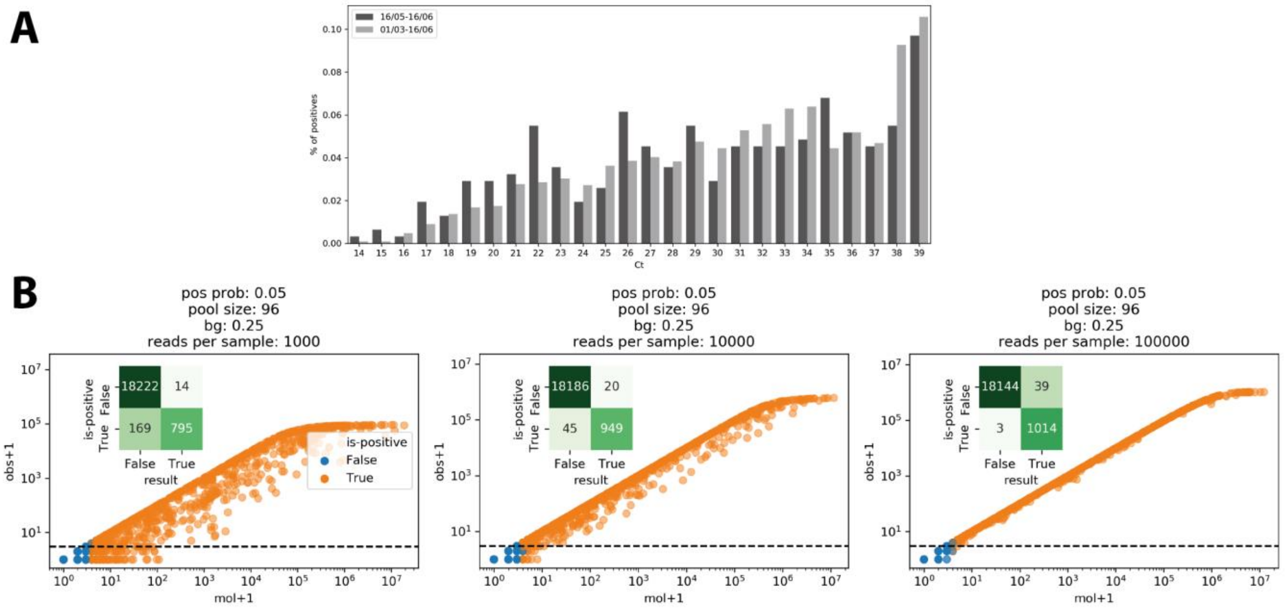

Supplementary Figure 5: Sequencing depth and FPR/FNR estimation

A) empirical Ct distribution obtained from the clinic in two different time periods.

B) Simulated Apharseq. Scatter plots of simulated samples in 96 sample batches (see note below). X-axis represents actual number of molecules in sample, Y-axis is the observed number of molecules, orange samples are positive, blue samples are negative, and the dashed line is the cutoff. Inset colored numbers are the confusion matrix - off diagonal entries are false negative (top right), and false positive (bottom left) samples. The different plots are with 1/10/100 thousand reads per sample (on average, from left to right).

Fig S6. RT primer contaminations in source plate

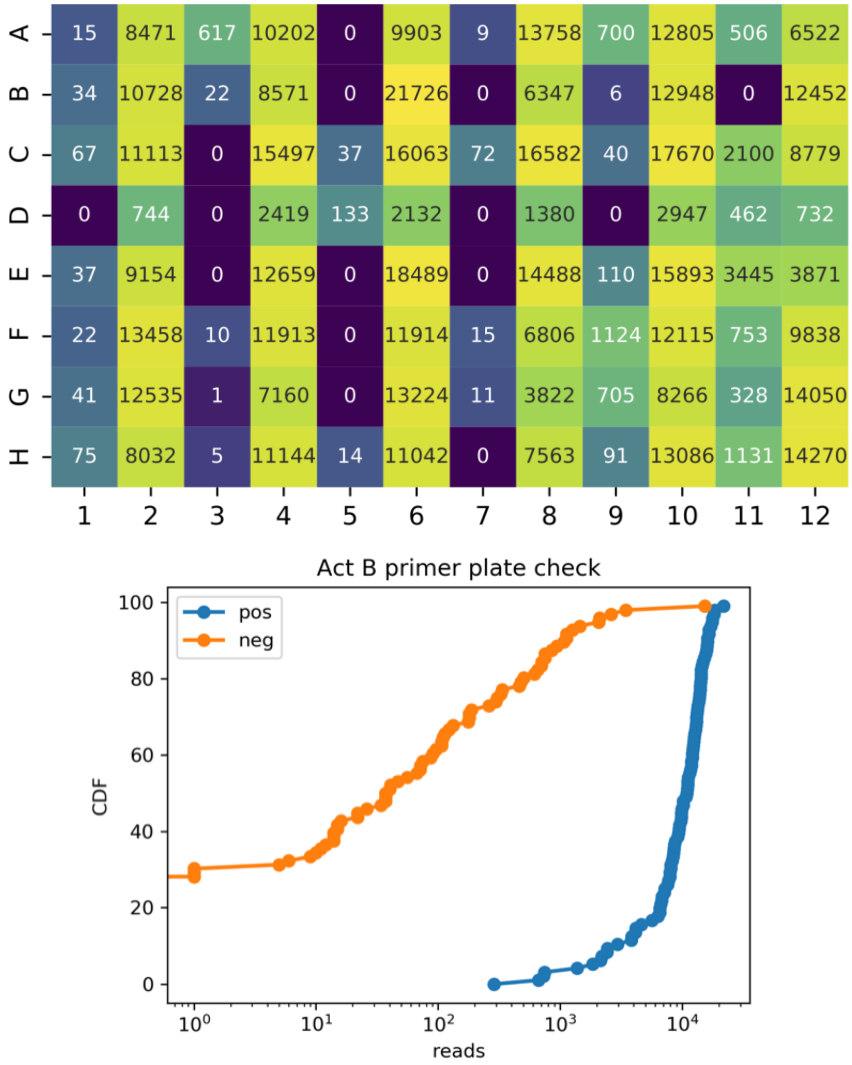

Supplementary Figure 6: RT primer contaminations in source plate

A) Barcoded ActB RT primers. Primers were collected from the even columns of the oligo plate as it arrived from the manufacturer into a single pool and used for RT and library preparation. There was noticeable contamination of oligos from the odd columns in the pool.

B) Positive/Negative cumulative distribution of the counts in S6A functions (number of reads per well, sorted from lowest to highest). This figure also includes the reciprocal test for odd columns of the plates.

Fig S7. Internal human control performance in ApharSeq

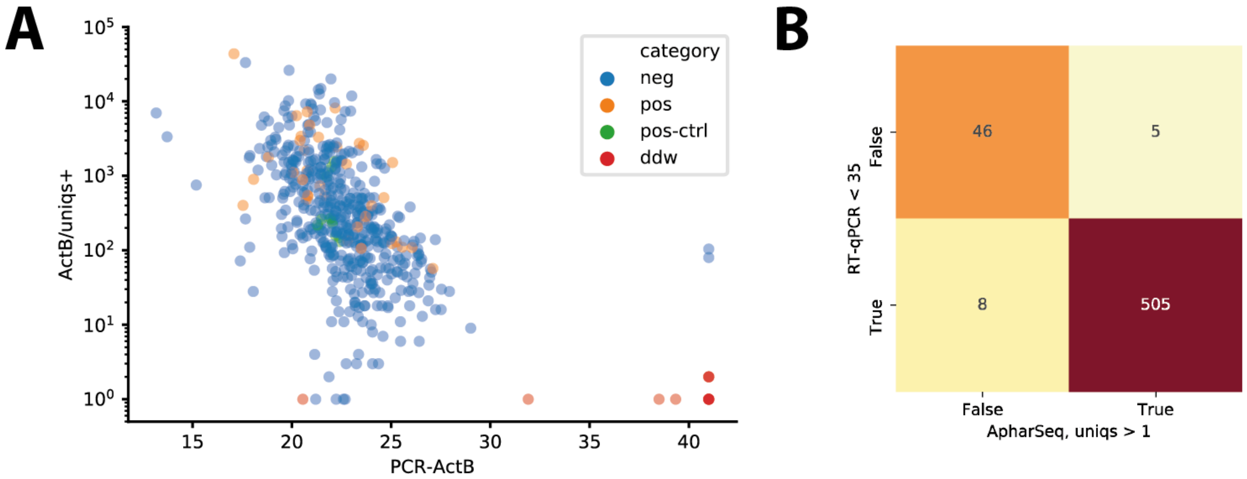

Supplementary Figure 7: Internal human control performance in ApharSeq

A) ActB Correlation between RT PCR signal (x-axis) and ApharSeq molecular counts (y-axis). Pearson r = -0.75 (p value < 2.8e-104).

B) Undetected ActB in samples (i.e. dropout samples) in both techniques is similar (5/8).

Supplementary Tables

Table S1. Experimental modifications Table.

All experiments follow the protocol published in Joseph-Strauss et al. *(45)*, with the following changes and specifications:

Fig.	bead vol [µl]	sample volume [µl]	lib. frac.	RT primers	Comments	Seq run	pK	Seq format (R1/R2/I1/I2)	internal name
2B	20	320	1	15 µl 100 µM	N/A	Tape	No	N/A	20-06-16_Neg_pools_Zymo_contamination
4C	20	320	1/30	15 µl 100 µM	rtPCR-primeScript kit: 25°C 10', 50°C 30'; 94°C 2'; 30 x {94°C 30'', 59°C 30'', 72°C 1'}; 72°C 10 min	15/6, 11/6	No	11/6: 150/0/8/8 15/6: 28/56/8/0	20-06-09_tube-titration
4B	20	320	1/2	15 µl 100 µM	2 step PCR: 10 cycles for first step, 20 cycles for extension PCR	2/6	No	38/38/8/8	20-06-02_tube-pools
3B	5	40	1/2	2 µl 10 µM	10 regular library PCR cycles followed by qpcr	qpcr	No	N/A	20-06-08_cross-contaminaion-qPCR
5A	13	320	1/2	15 µl 100 µM		21/6	No	36/40/8/8	20-06-02_tube-pools
5B	5	80	1/2	2 µl 10 µM	2 step PCR: 10 cycles for first step, 23 cycles for extension PCR	28/4	No	30/46/8/8	20-04-27_Human-Two_step_Lib
5C	20	320	1	15 µl 100 µM		24/6	No	80/0/8/0	20-06-22_ActB
3C	20	320	1	15 µl 100 µM		12/7	No	75/0/8/8	CHK_Primers_DWP_Act_N_E_Broad Ct
6	5	320	1/2	15 µl 10 µM	Buffer replacement and low-volume hybridization	25/8	No	20/8/8/56	ApharSeq_10Plates

Table S2. Required reagents/consumables and estimated costs.

Sample Collection		unit	per 100k	unit price	cost	percent
	VTM/UTM NP Swab (price from e.g. here)	1 box	250	640	160000	56.5
	DNA/RNA Shield x2 (Zymo R1200)	125 ml	140	290	40600	14.3
	2 ml deep well plate	20	50	102	5100	1.8
RNA cleanup and hybridization						

	polyT conjugated Sermag beads						
		Carboxyl coated beads from GE Healthcare, cat 65152105050450	1 L	0.1	25,000	2500	0.9
		5' amine modified 25 dT oligo from IDT	10 µmol	1	1980	1980	0.7
		EDAC (Sigma E7750)	1 g	1	52	52	0.0
		Imidazole (Sigma I2399)	100 g	0.14	85	11.9	0.0
		NaHCO3 (Sigma S8875)	500 g	0.05	33	1.65	0.0
		0.5M MES (Sigma 76039)	500 ml	0.2	1050	210	0.1
		Nuclease free water (Sigma W4502)	10 L	0.75	400	300	0.1
	Hybridization						
		Barcoded RT primers from IDT: ~70 bp oligo, standard desalting, 250 nmol plates x {N1/ActB}	plate pair	2	10,900	21800	7.7
		PCR plates	50 plates	20	235	4700	1.7
		DITI 150µL STERILE 3840 PCE. MCA96 SBS (Tecan 30038618)	40 box	26	480	12480	4.4
		8M LiCl (Sigma L7026)	1 L	2	345	690	0.2
		1M Tris-HCl, pH 7.5 (Sigma T2319)	1 L	3.2	110	352	0.1
		1M EDTA, pH 8.0 (Sigma 03690)	100 ml	6.4	55	352	0.1
		DTT (Sigma 43816)	250 ml	0.64	643	411.52	0.1
	Binding Buffer	Nuclease free water (Sigma W4502)	10 L	2.7	400	1080	0.4
	Wash A						
		2 ml deep well plate	50 plates	20	184	3680	1.3
		8M LiCl (Sigma L7026)	1 L	1	345	345	0.1
		1M Tris-HCl, pH 7.5 (Sigma T2319)	1 L	0.5	110	55	0.0
		1M EDTA, pH 8.0 (Sigma 03690)	100 ml	0.22	55	12.1	0.0
		20% SDS (Sigma 05030)	1 L	0.25	585	146.25	0.1
		Nuclease free water (Sigma W4502)	10 L	5	400	2000	0.7

	Wash B						
		8M LiCl (Sigma L7026)	1 L	0.02	345	6.9	0.0
		1M Tris-HCl, pH 7.5 (Sigma T2319)	1 L	10	110	1100	0.4
		Nuclease free water (Sigma W4502)	10 L	0.5	400	200	0.1
	Pool storage						
		RNALater (Sigma R0901)	500 ml	1	999	999	0.4
Enzymatic Reactions (per pool)							
	Proteinase K						
		Proteinase K (Epicenter MPRK092)	100 mg	4	225	900	0.3
		1M Tris-HCl, pH 8 (Sigma T3069)	1 L	0.001	296	0.296	0.0
		1M EDTA, pH 8.0 (Sigma 03690)	100 ml	0.005	55	0.275	0.0
		5M NaCl (Sigma 59222C)	1 L	0.003	107	0.321	0.0
		20% SDS (Sigma, 05030)	1 L	0.005	585	2.925	0.0
	Reverse Transcription						
		100 mM dNTP solution set (ThermoFisher R0181)	1 ml	2.5	144	360	0.1
		25 mM MgCl2 (Sigma M8787)	5 ml	2.4	139	333.6	0.1
		100% DMSO (Sigma D4540)	1 L	0.0025	221	0.5525	0.0
		Smart reverse transcriptase (Takara 639538)	400 rxn	2.5	1,000	2500	0.9
	SPRI Cleanup						
		Agencourt AMPure XP	60 ml	2.5	960	2400	0.8
		100% Ethanol	1 L	0.6	20	12	0.0
	PCR						
		Kapa HiFi DNA polymerase x2 mix (Kapa biosystems KK2612)	6.25 ml	4	300	1200	0.4
		PCR primers from IDT (~90/50 bp oligos, standard desalting)	100 nmol	60	0.6	36	0.0
Sequencing (Per pool of pools)							

	Quantification						
		Qubit dsDNA HS (ThermoFisher Q32851)	rxn	7		0	0.0
		HS DNA D1000 Tape (Agilent)	7 tapes	0.06	467	28.02	0.0
	Sequencing						
		NextSeq 92bp kit (Illumina 20024906)	kit	7	2,000	14000	4.9
total						283k	100.0
Excluding collection						82k	29.2

Table S3. DNA Oligos used in this study.

Provided as an external excel spreadsheet.
